# Study protocol for a multicentre comparative diagnostic accuracy study of tools to establish the presence and severity of peripheral arterial disease in people with diabetes mellitus: the DM PAD study

**DOI:** 10.1136/bmjopen-2022-066950

**Published:** 2022-11-03

**Authors:** Pasha Normahani, Laura Burgess, John Norrie, David Mark Epstein, Neghal Kandiyil, Athanasios Saratzis, Sasha Smith, Kamlesh Khunti, M Edmonds, Raju Ahluwalia, Trusha Coward, Tim Hartshorne, Simon Ashwell, Joseph Shalhoub, Elizabeth Pigott, Alun H Davies, Usman Jaffer

**Affiliations:** 1 Department of Surgery and Cancer, Imperial College London, London, UK; 2 Vascular Unit, Imperial College Healthcare NHS Trust, London, UK; 3 Usher Institute, Edinburgh Clinical Trials Unit, University of Edinburgh, Edinburgh, UK; 4 Edinburgh Clinical Trials Unit, Edinburgh, UK; 5 Faculty of Economics and Business Sciences, University of Granada, Granada, Spain; 6 Department of Radiology, University Hospitals of Leicester NHS Trust, Leicester, UK; 7 The Leicester Vascular Institute, University of Leicester, Leicester, UK; 8 Diabetes Research Centre, University of Leicester, Leicester, UK; 9 King's Diabetes Centre, King's College Hospital NHS Foundation Trust, London, UK; 10 Podiatry Services, Central London Community Healthcare Trust, London, UK; 11 Vascular Studies Unit, University Hospitals of Leicester NHS Trust, Leicester, UK; 12 Diabetes Care Centre, South Tees Hospitals NHS Foundation Trust, Middlesbrough, UK; 13 Patient co-investigator, London, UK

**Keywords:** diabetic foot, vascular surgery, vascular medicine, diabetic nephropathy & vascular disease, diagnostic radiology

## Abstract

**Introduction:**

Peripheral arterial disease (PAD) is a key risk factor for cardiovascular disease, foot ulceration and lower limb amputation in people with diabetes. Early diagnosis of PAD can enable optimisation of therapies to manage these risks. Its diagnosis is fundamental, though challenging in the context of diabetes. Although a variety of diagnostic bedside tests are available, there is no agreement as to which is the most accurate in routine clinical practice.

The aim of this study is to determine the diagnostic performance of a variety of tests (audible waveform assessment, visual waveform assessment, ankle brachial pressure index (ABPI), exercise ABPI and toe brachial pressure index (TBPI)) for the diagnosis of PAD in people with diabetes as determined by a reference test (CT angiography (CTA) or magnetic resonance angiography (MRA)). In selected centres, we also aim to evaluate the performance of a new point-of-care duplex ultrasound scan (PAD-scan).

**Methods and analysis:**

A prospective multicentre diagnostic accuracy study (ClinicalTrials.gov Identifier NCT05009602). We aim to recruit 730 people with diabetes from 18 centres across the UK, covering primary and secondary healthcare. Consenting participants will undergo the tests under investigation. Reference tests (CTA or MRA) will be performed within 6 weeks of the index tests. Imaging will be reported by blinded consultant radiologists at a core imaging lab, using a validated scoring system, which will also be used to categorise PAD severity. The presence of one or more arterial lesions of ≥50% stenosis, or tandem lesions with a combined value of ≥50%, will be used as the threshold for the diagnosis of PAD. The primary outcome measure of diagnostic performance will be test sensitivity.

**Ethics and dissemination:**

The study has received approval from the National Research Ethics Service (NRES) (REC reference 21/PR/1221). Results will be disseminated through research presentations and papers.

**Trial registration number:**

NCT05009602.

**STRENGTHS AND LIMITATIONS OF THIS STUDY**

- Multicentre study recruiting from primary, secondary and community healthcare will make the results generalisable.
- Pragmatic design and representative patient cohort will make the results immediately relevant to clinical practice.
- Study design has been heavily informed by patient and public involvement, thereby improving the chance of successful recruitment and completion.
- Reference test is not the traditional gold standard for peripheral arterial disease diagnosis (ie, intra-arterial angiography). Non-invasive, accurate cross-sectional arterial imaging with be the reference standard for this study.

## Introduction

Diabetes is a major global healthcare issue with an estimated prevalence of 9.3% (463 million people), rising to 10.2% (578 million) by 2030.^1^ Over 6% of people with diabetes develop a diabetic foot ulcer (DFU).^2^ DFUs are slow to heal,^3^ have a negative impact on patient quality of life^4^ and are associated with a 5-year lower limb amputation and mortality rate of 20% and 40%, respectively.^5^ In addition, DFUs cost the National Health Service (NHS) an estimated £1 billion per year.^6^

Peripheral arterial disease (PAD) is a key risk factor in the development of DFUs^7^ and is also associated with delayed DFU healing, increased risk of leg amputation and mortality.^3 8^ The detection of PAD in people with diabetes is fundamental, though challenging. Although a variety of bedside tests are available, there is no agreement as to which is the most useful.

### Existing evidence

Reviews of existing evidence^9–11^ highlight the lack of good quality evidence on this topic with a high risk of bias across studies, frequently relating to patient selection and lack of blinding.

The recently completed Testing for Arterial disease in Diabetes (TrEAD) study represents the largest study on this topic to date.^12 13^ The results of this study suggest that visual waveform assessment may be a promising modality. Furthermore, health economics modelling of the TrEAD data suggests that visual waveform assessment is the most cost-effective test (incremental cost-effectiveness ratio (ICER) £11 391), and that its use would result in a reduction in the number of amputations by 24% and cardiovascular deaths by 10% over 5 years as compared with next best alternative.^14^ However, these findings need to be further validated in a multicentre diagnostic accuracy study which addresses limitations relating to currently available evidence.

There are several important limitations relating to currently available evidence, which we aim to address in this proposed study. These include:

- Patient selection: No study has evaluated the full spectrum of the diabetic population seen in primary and secondary healthcare.
- Index and reference tests: Index tests in currently available studies were performed by expert staff whose experience may not represent the general healthcare workforce. All studies have used duplex ultrasound (DUS) as the reference test, which may be less reliable in interrogating the commonly affected distal vessels in those with diabetes^15 16^ as compared with CT angiography (CTA) or MR angiography (MRA).
- Analysis by limb: Most studies evaluated diagnostic performance by performing bilateral scans and interpreting results in each limb independently. This is a potential source of bias, as the presence of PAD in one limb increases the probability of PAD being present in the other.
- Visual waveform assessment: A significant arterial lesion results in morphological change in the waveform detected in the downstream circulation. Although visual waveform assessment has been shown to be a promising modality, there is currently no agreed definition of what constitutes an ‘abnormal’ waveform. Waveform morphology exists on a spectrum according to the severity of disease; triphasic (normal), biphasic and monophasic (abnormal). For the diagnosis of PAD, some studies use a monophasic cut-off,^17 18^ while others use a biphasic waveform as the threshold for diagnosis.^19^ The TrEAD study showed that overall test accuracy can be improved by using an enhanced definition for defining abnormal waveforms.^13^ This involves identifying biphasic waveforms with adverse morphological features, that is, spectral broadening, infilling of the spectral window, long forward flow or slow systolic rise time. This enhanced definition improved sensitivity as compared with the traditional monophasic waveform threshold (95% vs 77%), and improved specificity as compared with the biphasic waveform threshold (77% vs 21%). However, a potential limitation of the TrEAD study was that visual handheld Doppler assessment may have been disadvantaged by not using this enhanced definition, which was only evaluated for a new focused DUS test that directly visualised the ankle vessels (Podiatry Ankle Duplex scan; PAD-scan). In this proposed study, this enhanced definition, which has been shown to be superior, will be used as the primary diagnostic threshold for visual waveform assessment.

### Why this research is needed now

This research is of significant priority given the rising global prevalence of PAD^20^ and diabetes^21^ which will increase the burden of diabetic foot disease and place further pressures on healthcare services. Missed diagnosis of PAD is common,^22^ and is an important cause of avoidable amputations.^22–24^ Health economic modelling has demonstrated that improvements in the detection of PAD are not only cost-effective, but also may considerably reduce the number of lower limb amputations and cardiovascular deaths by enabling clinicians to optimise treatment.^14^ This will help mitigate the expected rise in disease (both PAD and diabetes) prevalence.

### Objectives

#### Primary objective

The primary objective of this study is to determine the diagnostic performance of index tests (audible handheld Doppler, visual handheld Doppler, ABPI, exercise ABPI and TBPI) for the diagnosis of PAD in people with diabetes as determined by a reference test (CTA or MRA).

#### Secondary objectives

- To determine the cost-effectiveness of tests over an appropriate time horizon of 5 years.
- To determine the performance of tests using exploratory diagnostic thresholds.
- To explore the effect of combining different tests on diagnostic performance.
- To evaluate patient acceptability of tests.
- To evaluate the effect of confounding patient characteristics (eg, neuropathy and ulceration) on diagnostic performance.
- To evaluate the performance of tests for establishing the severity of PAD.
- To evaluate inter-rater and intrarater reliability of tests.
- To evaluate the performance of PAD-scan (in selected centres).

## Methods and analysis

### Trial design

**Figure 1:**
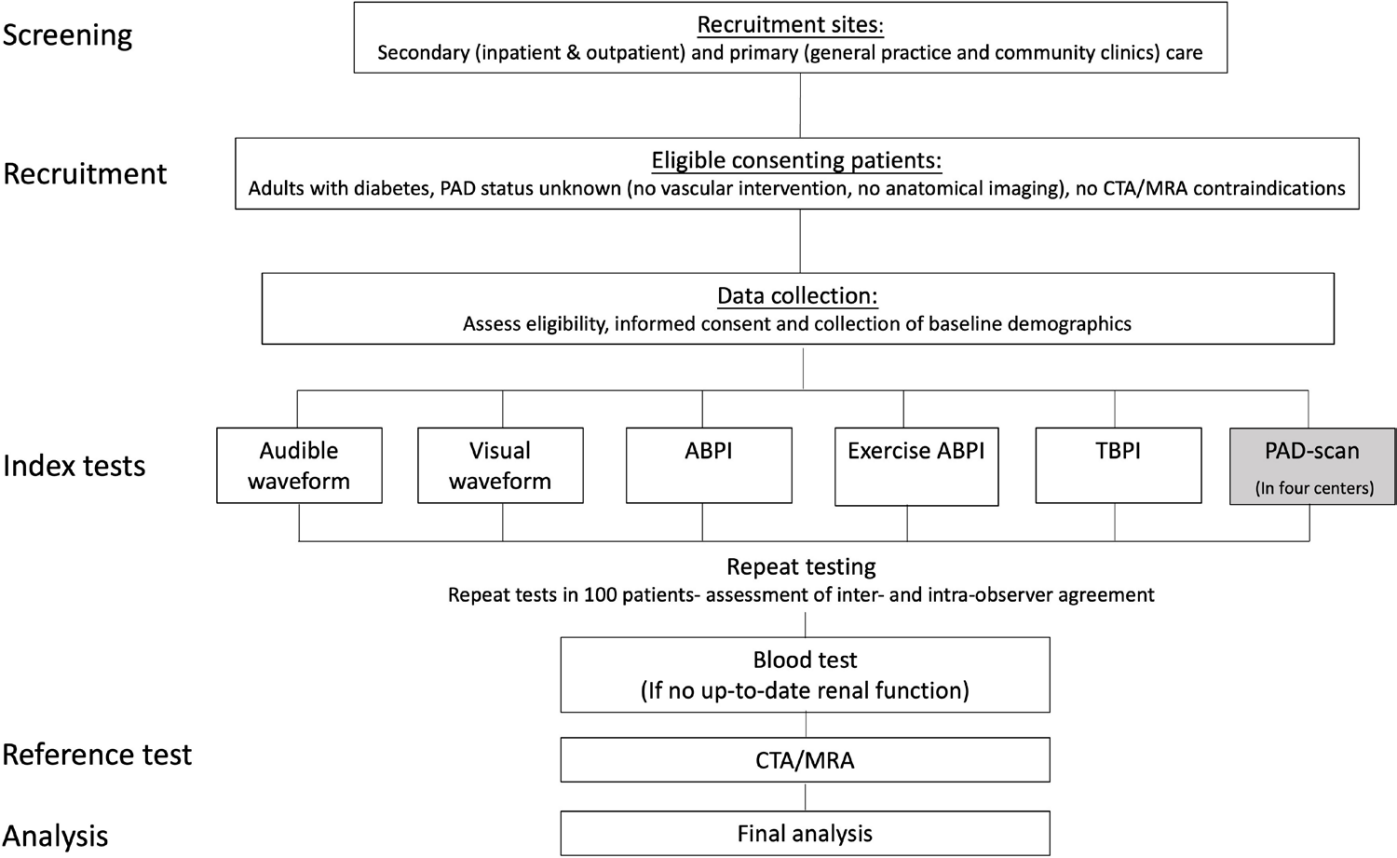
Study flow chart. ABPI, Ankle Brachial Pressure Index; CTA, CT angiography; MRA, MR angiography; PAD, peripheral arterial disease; TBPI, Toe Brachial Pressure Index.

This is a prospective comparative diagnostic accuracy study. The trial schema is presented in figure 1.

### Study setting

To ensure that all relevant healthcare settings are represented, we will be recruiting from community (n=2), primary (n=2) and secondary care (n=14). There will be 18 recruiting centres across the UK; London (n=4), South West (n=2), South East (n=3), East of England (n=1), East Midlands (n=1), West Midlands (n=1), Yorkshire and the Humber (n=1), North East (n=3), Wales (n=1) and Scotland (n=1).

### Eligibility criteria

The target population for this study are adults with diabetes, with or without DFU, presenting to vascular services (inpatient and outpatient), diabetic foot (community and secondary healthcare) and general practice clinics. Patients will be eligible for enrolment into the study if they fulfil the inclusion and exclusion criteria, as defined in box 1.

Box 1 **Inclusion and exclusion criteria**

**Inclusion criteria**

- Aged ≥18 years.
- Known history of diabetes.

**Exclusion criteria**

- PAD status known on imaging- prior knowledge may bias index tests.
- Known history of PAD intervention—prior knowledge may bias index tests.
- CTA and MRA contraindications—renal impairment, pregnancy, contrast medium hypersensitivity/allergy, non-compatible implants (MRA only).

### Recruitment

Recruitment will be primarily from vascular, diabetic foot and general practice clinics as well as inpatient wards. The start date of the study is April 2022 and we estimate that the study will complete within 12 months of commencement. This equates to 61 patients per month across 18 centres, that is, 3–4 patients per month per centre.

Adults with diabetes will be prescreened by a member of the direct care team. If eligible for recruitment and willing to speak to a research nurse. If willing, the study will be explained and if the patient gives verbal consent to receiving study information material (online supplemental appendix 1), these will be provided on visit 1, which coincides with a routine/planned visit. They will be told that if they agree to partake in the study and that, if they choose not to participate, this would not affect their usual clinical care. On visit 1, informed consent (online supplemental appendix 1) will be obtained before the participant undergoes any screening procedures. Following this, data will be collected and index tests completed. The visit schedule is summarised in table 1.

10.1136/bmjopen-2022-066950.supp1 Supplementary data

**Table 1:** Visit schedule

Visit no	Screening	Planned/routine visit	Blood test (in some centres)	Reference scan
	0	1	1b	2
Screening	X			
Study information material	X			
Informed consent		X		
Inclusion and exclusion criteria		X		
Demography		X		
Medical history		X		
Quality of life questionnaire (EQ-5D-5L)‡		X		
Index tests		X		
Repeat of index tests (same operator)*		X		
Repeat of index tests (alternative operator)*		X		
Blood test/pregnancy test		X	(X)†	
Reference scan (CTA/MRA)				X

*Repeat tests will only be performed in the first 100 volunteering patients.

†The blood test to assess renal function may require a separate, additional visit at some centres.

‡EQ-5D-5L, EuroQol- five-dimensional- five-level questionnaire

CTA, CT angiography; MRA, MR angiography.

### Data collection

A screening log will identify all approached patients and reasons for non-participation. The following data will be collected during visit 1:

- Demographics: age, gender, equality and diversity information, diabetes type, history of smoking, retinopathy, chronic kidney disease, ischaemic heart disease, stroke and heart failure.
- Foot history: PAD symptoms, previous history of DFU or amputation.
- Foot examination: neuropathy, presence of DFU, DFU severity using the WIfI classification system.^25^
- Technical success of index tests: inability to perform, refusal and discontinuation of tests will be documented.
- Results of index tests.
- Evaluation of patient acceptability: patients will be asked to rate their experience of each test on a Likert scale.
- Patient quality-of-life: EQ-5D-5L (EuroQol- five-dimensional- five-level) questionnaire.

### Interventions: index tests

Primarily we will be evaluating five index tests (ABPI, exercise ABPI, TBPI, visual handheld Doppler and audible handheld Doppler). In four centres, we will evaluate the PAD-scan as a sixth test. We have chosen not to evaluate this test at all centres, as DUS machines are moderately costly and not currently available at every site. The sites chosen to perform the PAD-scan have DUS machines available in their clinics to perform this.

All tests performed in clinic (or on the ward if the participant is admitted to hospital), during visit 1, by a member of the local clinical team so that results are generalisable. Tests will be performed on one limb; the most problematic side in symptomatic patients or a randomly selected side in asymptomatic patients. Tests and equipment will be standardised and team members will undergo protocol training.

Ideally, test order would be randomised to minimise influence carrying over from one test to the other. However, the audible and visual waveform tests involve semiobjective interpretation and therefore could be influenced by knowledge of the tests with an objective output (TBPI, ABPI and exercise ABPI). Therefore, semiobjective waveform tests will be performed first followed by the fully objective tests. Randomising the order of tests in these two blocks is not possible:

- Semiobjective tests: Audible waveform is less objective than visual waveform assessment and so should be performed first. However, in selected centres two forms of visual waveform assessment (handheld Doppler and PAD scan) are to be evaluated. The order of these two tests will be randomised (via REDCap) in these selected centres.
- Objective tests: TBPI should be performed before ABPI as it could be influenced by reactive hyperaemia secondary to proximal cuff inflation. Also, exercise ABPI should be performed last as exercise can influence all other tests.

**Figure 2:**
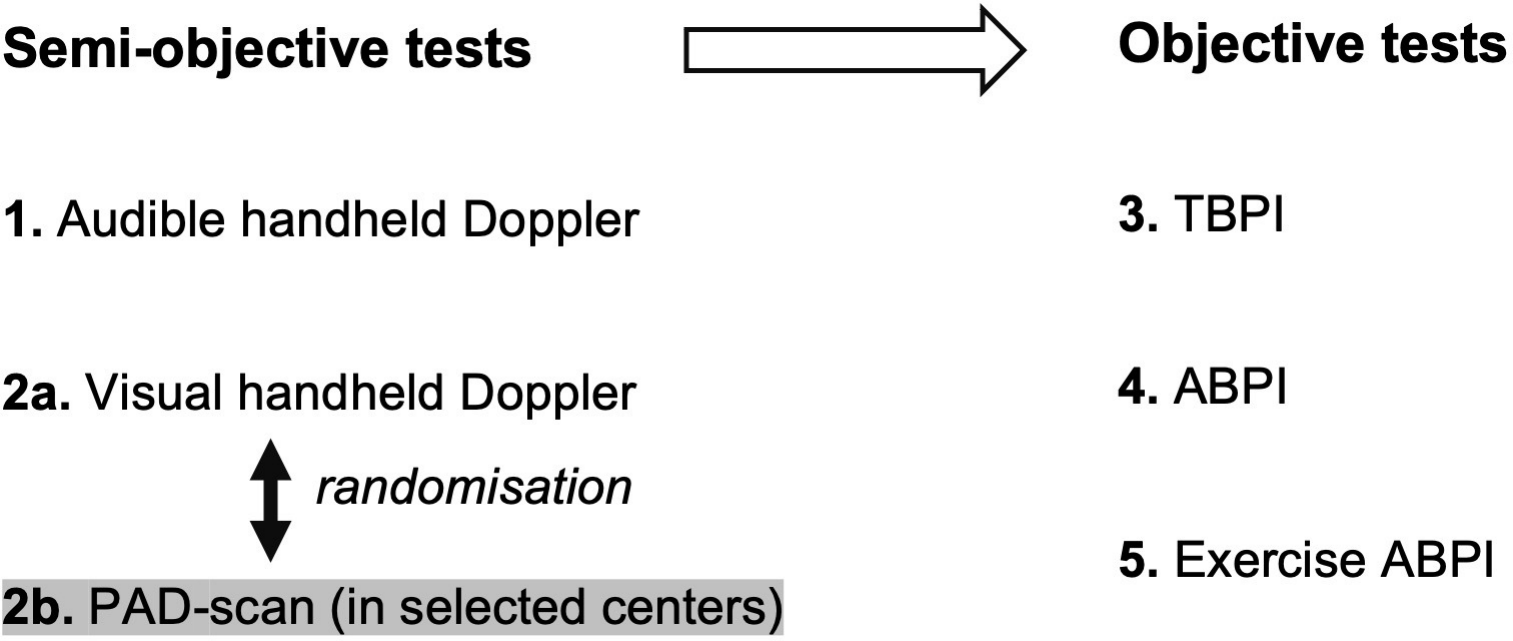
Order of index tests. ABPI, Ankle Brachial Pressure Index; PAD, peripheral arterial disease; TBPI, Toe Brachial Pressure Index.

The order of tests is summarised in figure 2.

#### Conducting index tests

Prior to conducting the first index test, participants will be rested in the supine position for at least 10 min with room temperature maintained between 23°C and 25°C. Details of index tests and diagnostic threshold are provided in online supplemental appendix 2.

#### Repeating index tests

The first 100 volunteering patients will have tests repeated on the same day by the same operator and also by another, blinded, operator for the assessment of intrarater and inter-rater reliability, respectively. Tests will be performed using the same descriptions outlined above. A minimum of 10 min rest must be provided to the patient prior to each batch of tests to avoid influence from previous tests carrying forward.

#### Reference test

Digital subtraction angiography (DSA) is considered the gold standard for the diagnosis of PAD. However, it is invasive and carries risks. Given the previously mentioned limitations of DUS our reference test will be cross sectional arterial imaging with CTA or MRA. Both have excellent accuracy compared with DSA.^26 27^ Some of our centres use only CTA, whereas others use only MRA. Additionally, some patients in our Patient and Public Involvement (PPI) survey reported that they would not take part if CTA was mandated and suggested the inclusion of MRA as an alternative.

Reference tests (CTA/MRA) will be performed according to a standardised protocol within 6 weeks of index tests. The final decision regarding whether the patient undergoes CTA or MRA will depend on local protocol and patient choice. Details of reference scan protocols can be found in online supplemental appendix 3. PAD is a chronic atherosclerotic condition and we do not envisage that there will be any change in disease status or reference test results over a 6-week period. Interim surgical interventions (occurring in the time interval between index and reference tests) will be considered a protocol violation and patients will be excluded.

Scans will initially be reported locally and then rereported centrally by a blinded consultant radiologist at the core lab (hosted by the University Hospitals of Leicester NHS Trust). Scans will be reported locally for identification of incidental abnormal clinical findings. Local reports will not be used as part of the study analysis. To assess inter-rater and intrarater reliability in the core lab, 15% of scans will be rereported by our core lab radiologists.

Scans at the core laboratory will be assessed using a validated angiographic scoring system (ANGIO score; online supplemental appendix 4)^28^; 10 major arteries supplying the lower limbs are each scored according to the degree of stenosis (0, 0%–49% stenosis; 1, non-occlusive stenosis of ≥50%; 2, complete occlusion). The presence of one or more arterial lesions of ≥50% stenosis will be used as threshold for the diagnosis of PAD. Tandem lesions with a combined value of ≥50% will also be considered positive for PAD as they are haemodynamically significant and in certain scenarios (eg, non-healing DFU) may prompt treatment. PAD severity will also be categorised according to the ANGIO score, as mild (≤4), moderate^5–9^ or severe (≥10). These categories have been shown to correlate with risk of amputation and cardiovascular events.^28^

### Outcomes

#### Primary outcome

Sensitivity of index tests.

#### Secondary outcomes

Specificity, likelihood ratios, predictive values and diagnostic OR.

- Health economic outcomes: (1) Cost of the test, including direct costs and amortisation of capital equipment and use of other healthcare resources for prevention and treatment of the disease over a time horizon of 5 years; (2) quality-adjusted life-yYears at 5 years and (3) ICER at 5 years.
- Patient acceptability (online supplemental appendix 5).
- Technical success.
- Inter-rater and intrarater reliability: The first 100 volunteering patients will be consented to have index tests repeated by the same operator and by an alternative operator on the same leg.

### Statistical analysis and plan

#### Sample size

Assuming a PAD prevalence of 50% (255 with PAD and 255 without PAD) the study will have 90% power to estimate an assumed sensitivity (or specificity) to a precision of the half width of the 95% CI of 8.2%. For a sensitivity (or specificity) of 80% this half width would increase to 10.2%. The level of significance was set at 1% to adjust for the five tests and ensure the overall level of significance does not exceed 5%. Power calculations used R.4.0.0 power.diagnostic.test in package MKmisc. The sample sizes for estimating likelihood ratios will also be estimated.

In the TrEAD study, PAD prevalence was 66%. As there will also be recruitment from primary care, with a lower PAD prevalence,^29^ the estimate to reflect the findings of our systematic review has been adjusted; the prevalence of 50% across 18 studies.^11^ In TrEAD, TBPI could not be performed in 20% of patients. A similar proportion may be unable to tolerate exercise ABPI. It is estimated that 10% of patients may drop out prior to the reference test. Therefore, the sample size has been inflated by the cumulative missingness across all groups (30%) to be certain of having enough power for each and every test comparison. Thus, we aim to recruit a total of 730 patients.

#### Sample size calculations for inter-rater and intra-rater reliability

In terms of sample size, an indicative calculation shows that using McNemar’s paired test on correlated proportions, with 100 participants, with no lost to follow-up, the study would have 90% power at a 5% level of significance to detect a difference of 0.17 in the discordant results (positive–negative vs negative–positive) between two tests (eg, 0.22 positive–negative vs 0.05 negative–positive).

#### Internal pilot

A stop-go assessment of recruitment feasibility will be included after a 4-month internal pilot. Recruitment feasibility will be assessed at the end of month 4, when 136 participants should have been recruited. If 90 or fewer have been recruited, the study may be stopped (RED); between 90 and 114 adapt (AMBER—more sites and/or more time) and if 115 or more continue unchanged (GREEN—within sampling variability of our target). This will be discussed with the trial steering committee (TSC) and the funder.

#### Statistical analysis

The five individual tests (and the sixth exploratory test in four sites) will be compared against the reference test (CTA/MRA), calculating standard diagnostic accuracy metrics of sensitivity, specificity, predictive values, likelihood ratios and diagnostic OR (using the bivariate model approach implemented in R). A 95% CIs calculated at 99% to adjust for the five comparisons will be presented. The robustness of the findings to any observed patterns of missing data will be assessed, which are expected to differ by test. A multiple imputation approach will be used, assuming the data are missing at random. In addition, and probably more consistent with the likely missing data generating mechanisms, sensitivity type analyses assuming the data are missing not at random (ie, informatively missing) will be explored. This would attempt to identify different types of missing data by an underlying reason or reasons, and then imputing values that capture plausible measurements for those missing data. The delta adjustment approach given by van Buuren will be followed^30^ and also the recommendations of Molenburghs and Kenward.^31^ These approaches would allow the set of reasons for missing values to vary across the tests. The purpose is to stress the calculated findings to test their robustness to the observed patterns of missing data.

The subgroups of disease severity (both clinically and radiologically defined as detailed below) will be explored and those with/without neuropathy or DFU. The subgroups in the Statistical Analysis Plan (SAP) will be prespecified. Any further subgroup analysis (eg, if suggested later by new data external to the study) will be labelled as exploratory. Prespecified subgroup analyses will be unlikely to be adequately powered. Clinical severity will be graded according to the severity of symptoms (from least to most severe; asymptomatic, intermittent claudication, rest pain and tissue loss). Severity will be measured radiologically using the ANGIO-score as outlined. Both will be analysed as prespecified subgroup analyses in the SAP.

Combinations of tests will be explored to see if using more than one test has incremental diagnostic value. The combinations of tests that were clinically felt to potentially offer an improvement over individual tests will be prespecified in the SAP and then, acknowledging the paired data, use the approach of Pepe and Thomson,^32^ which looks at linear combinations of the underlying tests. Post hoc checks will be made if there were combinations that were not prespecified that performed even better, as hypotheses for subsequent evaluation.

It is important to quantify the ability of each of the five index tests to measure consistently the same measurement of interest on the same leg of the same subject using the same test kit in the same location and the same environmental conditions, within a short period of time. This quantification of the intrarater repeatability (or reproducibility) will be undertaken using the test–retest approach.^33 34^ The inter-rater reliability (the agreement between two or more clinicians measuring the same subject, again as under the conditions above) using appropriate methodology^33 34^ will be quantified. For the inter-rater and intrarater repeatability, we will aim for a sample size of 100 per a pair of index tests.

These reliability studies will be performed at the start of the study and analysed as soon as the data are mature. If an index test has unacceptable intrarater repeatability, or unacceptable inter-rater reliability, it could be dropped from further consideration, following discussions with the independent TSC. Unacceptable intrarater and inter-rater reliability will be assessed in two ways. First, in an absolute sense, by looking at the kappa statistics and using the published guidance as to what an acceptable magnitude is,^35^ with a kappa of <0.4 considered unacceptable. There is no unanimity over interpreting the magnitude of kappa statistics, so our second approach will compare the kappa statistics across the tests and label unacceptable any tests that are substantially worse than the other tests.

Inter-rater and intrarater reliability will also be assessed for the reporting of reference tests using the methods outlined above. Reference tests will not be repeated due to feasibility and ethical considerations.

Full details of the methods and justification of the sample sizes will be included in the comprehensive SAP, authored by the study statistician and agreed by the independent TSC. The SAP will be prepared and finalised prior to database lock.

#### Health economics analysis

The health economic analysis aims to assess the likely impact of a more accurate diagnostic test on treatment choices, health outcomes that are important to patients (namely DFU incidence and healing time, cardiovascular events, amputations and mortality), and the impact on use of national healthcare system resources of testing, preventative interventions and treatments of disease. As there will not be clinical follow-up in the DM PAD study to determine these outcomes, these questions will be addressed by modelling methods that simulate clinical events that would occur in these patients under different counterfactual testing options. A literature review will be conducted to identify published health economic studies in similar patient groups. The structure and evidence that will be used in constructing the model will be determined following this review but may follow and update previous work by this group.^14^ Depending on ulceration status at presentation, this model classified patients into one of eight initial states following a test: true positive (with and without DFU), true negative (with and without DFU), false positive (with and without DFU) or false negative (with and without DFU). It was assumed that true and false positive patients without DFU would be prescribed orthotics and additional foot checks, in addition to standard care. True and false positive patients with DFU would undergo confirmatory imaging and, if confirmed positive, angiography, revascularisation and low-dose rivaroxaban, in addition to standard care. True and false negative patients would continue with standard care for the remainder of the 5-year time horizon. The probability of clinical events (new DFU incidence and healing rates of DFU, amputation of unhealed limbs, cardiovascular events and death) and treatment effects associated with recommended interventions for diagnosed PAD patients (eg, orthotics, revascularisation, rivaroxaban) were obtained from national evidence reviews and the literature.

The study will be conducted from the perspective of the UK NHS and Personal Social Services according to established methodological guidelines and reporting standards.^36^ Prices and unit costs of healthcare resources will be obtained from manufacturers and national databases.

### Data management

Data will be written directly into the case report form (CRF) (source data) and then transcribed into the electronic CRF. Source documents include original documents related to the trial, to medical treatment and to the history of the participant, and adequate source documentation will be maintained to allow reliable verification and validation of the trial data.

Data management will be through REDCap, a web-based data entry system and database. The data management services team (based at Edinburgh Clinical Trials Unit) will work with the investigators, trial Manager, trial statisticians and trial teams to design and build bespoke electronic CRFs and validation rules for data entry to ensure that the data are collected accurately and stored securely. They will also provide the appropriate user training. The trial manager will visit the sites to verify the quality of the data.

### Study management

The organisational structure for the study, including details of monitoring procedures, quality control and assurance, is outlined in online supplemental appendix 6.

### Patient and public involvement

The development of the proposal has been informed by patients and the public.

#### Learning from the experience of patients in the TrEAD study

A telephone survey of 57 patients from the TrEAD study followed by a focus group discussion was conducted. The strength of this approach is that the PPI is not centred around hypothetical discussions but incorporates the perspective of patients involved in a similar study. This informed the following changes:

- Incorporating a non-treadmill exercise ABPI test (see index tests’).
- Advertising the study online to improve accessibility for all patients (see ‘equality, diversity and inclusion’).
- Providing a lay summary of individual test results with actionable recommendations, in addition to the general practitioner letter (see ‘dissemination, engagement and projected outputs’).
- Patients anticipated difficulties in accessing different parts of hospitals for blood tests and imaging, due to difficult directions and access issues for those with disabilities. After discussion, it was agreed that we should work with local sites to ensure that clear written directions are made available to patients and blood tests are performed, where feasible, at the same location as index tests.

#### Learning from the wider diabetic community

An online survey of 123 people was conducted; 96% felt the research was important. 6% indicated that they would not take part if CTA was necessitated, and 10% felt the study was not easy to understand. This prompted us to make the following changes, which were accepted by our study focus group:

- Include MRA as an alternative reference imaging modality.
- Incorporate information regarding CTA radiation exposure with a ‘real-world’ comparison in our patient information sheet.
- Drafting and revising our ‘Plain English Summary’.

### Ongoing PPI

Our patient coinvestigator, EP will chair a patient advisor group who will meet annually to ensure that a wide range of patient perspectives are considered during the study. The patient advisor group will also contribute to the interpretation of study findings, thereby allowing us to integrate patients’ perspectives in the analysis phase.

## Ethics and dissemination

### Ethics

The study has received approval from the National Research Ethics Service (NRES) London Central Committee (Reference 21/PR/1221). The study has been registered on ClinicalTrials.gov (Registration number NCT05009602).

### Dissemination plan

Results will be reported according to the Standards for Reporting of Diagnostic Accuracy Studies checklist.^37^ Results will be disseminated through scientific conferences and peer-reviewed publications. Study participants and relevant patient support groups will be informed of the study results.

## Supplementary Material

Reviewer comments
