# Supplementary material for: Study protocol for a multicentre comparative diagnostic accuracy study of tools to establish the presence and severity of peripheral arterial disease in people with diabetes mellitus: the DM PAD study
Source: BMJ Open. 2022 Nov 3;12(11):e066950. doi: 10.1136/bmjopen-2022-066950 (PMC9639108; doi:10.1136/bmjopen-2022-066950)
Supplement: Supplementary data [file bmjopen-2022-066950supp001.pdf]

BMJ Publishing Group Limited (BMJ) disclaims all liability and responsibility arising from any reliance placed on this supplemental material which has been supplied by the author(s)

SUPPLEMENTARY MATERIAL

Contents

APPENDIX 1- PATIENT INFORMATION SHEET AND INFORMED CONSENT DOCUMENT 2

APPENDIX 2- DETAILS OF INDEX TESTS 20

APPENDIX 3- REFERENCE CTA AND MRA PROTOCOL 23

APPENDIX 4- ANGIO SCORE 25

APPENDIX 5- EVALUATION OF PATIENT ACCEPTABILITY LIKERT SCALE 26

APPENDIX 6- STUDY MANAGEMENT STRUCTURE 27

Normahani P, *et al. BMJ Open* 2022; 12:e066950. doi: 10.1136/bmjopen-2022-066950

BMJ Publishing Group Limited (BMJ) disclaims all liability and responsibility arising from any reliance placed on this supplemental material which has been supplied by the author(s)

Appendix 1- Patient information sheet and informed consent document

Delete this line, then print on Hospital/Trust headed paper

DM PAD

A multicenter comparative diagnostic accuracy study

Diagnostic tools to establish the presence and severity of peripheral arterial disease in people with diabetes (DM PAD)

ISRCTN: 12358788

HRA/REC Reference: NIHR131855

IRAS: 301408

Sponsor Reference: 21CX7046

Acknowledgement

This project is funded by a National Institute for Health Research, Health Technology Assessment programme grant, which is funded by the National Institute for Health Research (NIHR)

Disclaimer

The views expressed in this publication are those of the author(s) and not necessarily those of the MRC, NHS, NIHR or the Department of Health.

PATIENT INFORMATION SHEET AND INFORMED CONSENT DOCUMENT

You have been invited to take part in a research study called DM PAD. Before you decide whether to accept, we would like to explain why the research is being carried out and what it will involve.

Please read this information carefully, and discuss it with others if you wish. Ask us if anything is unclear, or if you would like more information.

- *Part 1 tells you the purpose of this study and what will happen to you if you take part.*
- *Part 2 gives you more detailed information about the conduct of the study.*

Please take your time to decide whether or not you wish to take part.

Thank you for reading this information sheet.

Normahani P, *et al. BMJ Open* 2022; 12:e066950. doi: 10.1136/bmjopen-2022-066950

<u>Part 1</u>

What is the purpose of the study?

In the UK there are over 7,000 leg amputations each year because of diabetes. The most important cause of this is poor circulation. The detection of poor circulation in patients with diabetes is difficult. A number of tests exist to detect poor circulation (known as peripheral arterial disease). However, there is confusion as to which is the gold standard. We propose to carry out this study to determine which test is the most accurate.

Why have I been chosen?

You have been invited to consider this study because you are a patient with diabetes. We hope that about 730 people like you from across the UK will take part in this study.

Do I have to take part?

No, participation in this study is entirely voluntary. If you do decide to take part you will be given this information sheet to keep. You will be asked to sign a consent form, but you are still free to withdraw at any time and without giving a reason. If you decide not to take part in the study, your doctor will be happy to talk through how he/she will treat you outside of the study. You do not have to give a reason for not taking part and your treatment and care will not be affected in any way.

What will happen to me if I take part?

If you choose to be involved in the research, your participation will last 6 weeks from trial entry. Although the study is conducted over 2 visits, in most cases, the first visit will be conducted at the time of a routine visit that you already have scheduled. Therefore, you will only be required to make one additional visit. In some centres, an additional visit may be required to take a blood sample prior to the imaging scan (i.e. 3 visits in total). Your doctor or nurse will inform you if this is the case. We have designed the study in this way to make it as easy as possible for you to take part.

If you are currently an inpatient, your first visit will be conducted at your bedside. Where possible, if you remain an inpatient, your second visit scan will also be performed during your inpatient stay to avoid additional hospital visits.
The following section tells you more about what will happen on each of your visits.

There are no restrictions on your activity when you are taking part in this study. You will continue with any other medical care or treatments, such as taking regular medication, as you would normally do. This will include the standard treatment for diabetes, which may include dietary advice, blood glucose management and drug treatment.

Your first visit

If you decide to participate in the study your first visit will coincide with a routine visit that you will have booked as part of your standard care, irrespective of whether you wish to participate in the DM PAD study. The following steps will be taken at your first visit, which will last approximately 1 hour 10 minutes (i.e., 40 minutes longer than your routine scheduled appointment):

- You will first be asked to sign the consent form to confirm that you would like to be included (you will be given a copy of this).
- Some information about your medical history and current medical condition will be collected to check that you are able to take part.

If the research team confirm that you are able to take part in the study, you will have your blood pressure and pulse rate measured. Other measurements that will be recorded include height, weight and body mass index. We will also ask some questions regarding demographic details, including your gender and ethnicity.

At this visit you will also be also asked to complete a health questionnaire about your quality of life so we can collect information about your health. This is called the EQ-5D-5L questionnaire.

As part of your routine care, a doctor or nurse will perform an assessment for neuropathy and, if relevant, assess the severity of your foot ulcer. A blood test to assess your renal (kidney)

function will also be taken in preparation for visit 2. This blood test may require a separate, additional, visit at a different site. Your doctor or nurse will inform you if this is the case.

A total of five different tests will be performed on either one (visible and audible blood flow) or both (blood pressure measurements) legs. A number of these tests are routinely performed, others, marked with an asterisk (*) are additional tests that are being evaluated in this study:

1. Blood pressure measurements at the arm and ankle;
2. Blood pressure measurements at the arm and ankle following repetitive heal raises*;
3. Blood pressure measurements at the big toe*;
4. Visible blood flow waveform (using a handheld Doppler)*;
5. Audible blood flow waveform (using a handheld Doppler);

The visible and audible waveform assessments involve a handheld Doppler device to check for the presence or absence of a signal in the arteries in the leg. The visual blood flow waveforms will be saved using a code that means you cannot be directly identified (‘pseudonymised’), and may be used for future analysis”

For each of the tests, you will be asked to rate your experience.

In certain centres, a sixth test will be performed, called a Podiatry Ankle Duplex scan (PAD-scan)* on one leg. This is a new ultrasound test that directly visualises the ankle vessels and detects more detailed waveforms.

The visible and audible handheld Doppler tests, as well as the PAD-scan, are very similar. They all involve the brief application of a sensor and gel on the skin to assess the quality of blood flow to the foot.

A sub-group of 100 patients will be asked to consent to having all the above index tests repeated (in the same leg) on the same day, by the same operator and also by an alternative operator to assess reliability. This will take an additional 40 minutes.

Your second visit

The second visit will take place within 6 weeks of the first visit and may take place at a different participating site if you were recruited into the study at a primary care site or in the community (due to access to imaging equipment). Your doctor or nurse will inform you if this is the case. In the second visit, which will take 30 minutes, you will have a more detailed scan of your blood vessels in your leg using a computed tomography angiography (CTA) or magnetic resonance angiography (MRA) scan. If you were to choose not to take part in this study there would still be a 2 out of 10 (20%) chance that you would still undergo one of these tests for further evaluation. The results from the index tests will be compared to the results of the CTA or MRA to identify the most accurate test.

Your doctor or nurse will inform you of which scan (CTA or MRA) will be performed prior to your appointment. In some centres, both scans may not be available as an option.

If you are on metformin, you may be advised to withhold this before the scan for a short period of time.

CTA

CTA uses x-rays with detectors and a powerful computer to produce two-dimensional and three-dimensional images of the blood vessels.

An injection of a contrast dye will be given into a vein in the arm to highlight blood vessels. You will lie on your back in the scanner. You must hold your breath and remain still while the scans are taken. The contrast dye injection gives a sensation of warmth, and sometimes a metallic taste in the mouth which is normal – this rapidly clears.

The images are then processed by a powerful computer which can then display the arteries clear of the soft tissues around them to give an accurate diagnosis of any disease of the arteries.

The scan will last approximately 5-10 minutes in total.

CTA is associated with some radiation exposure. More information on this is provided later in this information sheet.

MRA

MRA is a type of MRI scan which uses powerful magnets, radio waves and computers to generate images of the body, specifically to look at the blood vessels. To get best views, it is usually necessary to give some contrast medium into your vein at the time of the scan. The scanner is a large tube which you lay inside on a scan table. The table moves through the scanner to take images of your body. You will need to lie as still as possible. During the scan you will hear loud knocking noises, which occur in bursts during each imaging sequence.

The scan usually takes about 15 - 20 minutes.

MRA scans may not be recommended in certain situations. For example, if you have a metal implant fitted such as a pacemaker, internal cardiac defibrillator, cochlear implants in your ear, a nerve stimulator, surgical clips or any implants).

What is the standard treatment in the UK?

If you decide not to take part in the trial, in most hospitals you will be offered the standard care for diabetes which includes dietary advice, blood glucose management and drug treatment.

Unwanted effects of treatment

You should not join the study if you are pregnant (or plan on becoming pregnant during the course of the study), have renal impairment, have a known hypersensitivity/allergy to contrast medium, or have non-compatible implants (for patients undergoing an MRA only).

Pregnancy

CTA and MRA scans are contraindicated in pregnant patients. Pregnant women must not therefore take part in this study; neither should women who plan to become pregnant during the study. Women who could become pregnant will be asked to have a pregnancy test before taking part to exclude the possibility of pregnancy and must use an effective contraceptive during the course of this study. Contraceptive methods that can achieve a failure rate of less than 1% per year and when used consistently and correctly are considered as highly effective birth control methods. These include:

- ✓ combined (estrogen and progestogen containing) hormonal contraception associated with inhibition of ovulation:
  - oral
  - intravaginal
  - transdermal
- ✓ progestogen-only hormonal contraception associated with inhibition of ovulation:
  - oral
  - injectable
  - implantable
- ✓ intrauterine device (IUD)
- ✓ intrauterine hormone-releasing system (IUS)
- ✓ bilateral tubal occlusion
- ✓ vasectomised partner
- ✓ sexual abstinence- if this is a compatible lifestyle choice

Any woman who finds that she has become pregnant while taking part in the study should immediately tell her research doctor. The research team may request to collect additional follow up information in such circumstances.

You may be withdrawn from the study if the doctor feels it is best for you not to participate or if you do not comply with the requirements of the study. If during the health screening tests any abnormal results are found, you will be immediately referred for clinical review as appropriate. If you feel any discomfort or distress during the study you must say so immediately and we will stop straight away. If for any reason during the study and you do not

wish to continue, than we will stop the tests immediately.

How is my condition monitored?

Participating in this study will not significantly affect how your condition is monitored or any other treatment you receive.

What are the possible benefits of taking part?

If you decide to participate, you will undergo comprehensive testing and imaging for peripheral arterial disease (poor circulation) as part of this study. If previously undiagnosed, the diagnosis of peripheral arterial disease may prompt your doctor to initiate medicines that will help reduce your chance of having a stroke, heart attack and worsening of your peripheral arterial disease.

Additionally, if you are diagnosed as having peripheral arterial disease, you may be eligible for additional measures to reduce the risk of developing a foot ulcer. These additional measures may include more frequent foot checks or new footwear.

If you have an active ulcer, a diagnosis of peripheral arterial disease will trigger a specialist review by a vascular surgeon for consideration of timely revascularisation to improve the chance of ulcer healing and reduce the risk of amputation.
You will not get paid for participating in this study but can claim for travel expenses.

What are the possible disadvantages and risks of taking part?

If you take part in this study you may have a Computed Tomography Angiography (CTA) procedure, which, for the majority of participants (about 80%), will be additional to the procedures you would have if you did not take part. This procedure uses ionising radiation to form images of your body and provide your doctor with other clinical information. Ionising radiation may cause cancer many years or decades after the exposure.

We are all at risk of developing cancer during our lifetime. 50% of the population is likely to develop one of the many forms of cancer at some stage during our lifetime. Taking part in this study may increase the chances of this happening to you to about 0.12% or 1 in 800.

CTA should NOT be used by the following individuals:

- pregnant women
- patients with renal impairment
- patients with a known hypersensitivity/allergy to contrast medium
- patients fitted with non-compatible implants (for patients undergoing an MRA only).

What happens when the research study stops?

The information from this study will be used to decide the most accurate test for the diagnosis of peripheral arterial disease in diabetic patients.

At the end of the trial you will revert to standard care for your condition.

Will my taking part be kept confidential?

Yes, it will. If you decide to participate, the information collected about you will be handled strictly in accordance with the consent form that you have signed and also the 2018 Data Protection Act. Please refer to Part 2 for further details.

This completes Part 1 of the Information Sheet. If the Information in Part 1 has interested you and you are considering participation, please continue to read the additional information in Part 2 before making any decision.

Part 2

What if relevant new information becomes available?

Sometimes during the course of a research project, new information becomes available about a treatment that is being studied. If this happens, your research doctor will tell you about it and will discuss with you whether you want to continue in the study. If you decide to withdraw your research doctor will make arrangements for your care to continue. If you decide to continue in the study you will be asked to sign an updated consent form.

What will happen if I don't want to carry on with the study?

You can decide to leave the study at any time. You do not need to give a reason.

If you leave the study before your treatment, then your doctor will discuss with you what type of treatment they will use outside the study. If you decide to leave the study, any data collected up until that time will remain on file and will be included in the final study analysis and follow up information will continue to be collected from your medical records.

If you decide to leave the study and do not wish for any further data to be collected about you, you should inform your clinical care team of this in order that no further follow up information is collected from your medical records. In line with Good Clinical Practice guidelines, at the end of the study, your data will be securely archived. In this study, data will be archived for a minimum of 10 years after which arrangements for confidential destruction will be made.

What if something goes wrong?

A group of independent researchers (called the Data Monitoring Committee) will closely monitor the study. If there are any problems then they will be detected as soon as possible so that the study can be changed or stopped if necessary.

Imperial College London holds insurance policies which apply to this study. If you experience harm or injury as a result of taking part in this study, you will be eligible to claim compensation without having to prove that Imperial College is at fault. This does not affect your legal rights to seek compensation.

If you are harmed due to someone's negligence, then you may have grounds for legal action. Regardless of this, if you wish to complain, or have any concerns about any aspect of

the way you have been treated during the course of this study then you should immediately inform the Investigator (Insert name and contact details). The normal National Health Service complaints mechanisms are also available to you. If you are not satisfied with the response, you may contact your local Patient Advice and Liaison Service (PALS) which offers confidential advice, support and information on health-related matters (insert contact details). You may also contact the Imperial AHSC Research Governance and Integrity office (Room 215, Medical School Building, St. Marys Campus, Norfolk Place, London W2 1PG. Tel: 0207 594 9459).

How will we use information about you?

Imperial College London is the sponsor for this study and will act as the data controller for this study. This means that we are responsible for looking after your information and using it properly. Imperial College London will keep your personal data for:

- 10 years after the study has finished in relation to data subject consent forms.
- 10 years after the study has completed in relation to primary research data.

We will need to use information from you, your medical records and possibly your GP for this research project.

This information will include your initials, NHS number, name and contact details such as telephone number and address. People will use this information to do the research or to check your records to make sure that the research is being done properly.
People who do not need to know who you are will not be able to see your name or contact details. Your data will have a code number instead.
We will keep all information about you safe and secure.

Some of your information will be sent to the University of Granada based in Spain, and the University Hospitals of Leicester NHS Trust based in the United Kingdom. They must follow our rules about keeping your information safe.

Once we have finished the study, we will keep some of the data so we can check the results. We will write our reports in a way that no-one can work out that you took part in the study.

Normahani P, *et al. BMJ Open* 2022; 12:e066950. doi: 10.1136/bmjopen-2022-066950

Legal basis

As a university we use personally-identifiable information to conduct research to improve health, care and services. As a publicly-funded organisation, we have to ensure that it is in the public interest when we use personally-identifiable information from people who have agreed to take part in research. This means that when you agree to take part in a research study, we will use your data in the ways needed to conduct and analyse the research study.

Health and care research should serve the public interest, which means that we have to demonstrate that our research serves the interests of society as a whole. We do this by following the UK Policy Framework for Health and Social Care Research

International transfers

There may be a requirement to transfer information to countries outside the European Economic Area (for example, to a research partner). Where this information contains your personal data, Imperial College London will ensure that it is transferred in accordance with data protection legislation. If the data is transferred to a country which is not subject to a European Commission (**EC**) adequacy decision in respect of its data protection standards, Imperial College London will enter into a data sharing agreement with the recipient organisation that incorporates EC approved standard contractual clauses that safeguard how your personal data is processed.

Sharing your information with others

For the purposes referred to in this privacy notice and relying on the bases for processing as set out above, we will share your personal data with certain third parties.

- Other College employees, agents, contractors and service providers (for example, suppliers of printing and mailing services, email communication services or web services, or suppliers who help us carry out any of the activities described above). Our third party service providers are required to enter into data processing agreements with us. We only permit them to process your personal data for specified purposes and in accordance with our policies.
- the following Research Collaborators / Partners in the study;

- University of Granada - the Health Economist is based at the University of Granada and therefore your de-identified health economic and quality of life data will be transferred there.
- University Hospitals of Leicester NHS Trust - the scan you have performed at your second visit (CTA or MRA) will be reported by a radiologist at University Hospitals of Leicester NHS Trust and therefore your medical scan images will be transferred there via a secure NHS system.
- University of Edinburgh - with your permission, your data will be entered onto a secure database held at the University of Edinburgh, in accordance with the 2018 Data Protection Act.

What are your choices about how your information is used?

You can stop being part of the study at any time, without giving a reason, but we will keep information about you that we already have. We need to manage your records in specific ways for the research to be reliable. This means that we won't be able to let you see or change the data we hold about you.

If you agree to take part in this study, you will have the option to take part in future research using your data saved from this study.

Anonymous data may also be linked with appropriate national databases, including Hospital Episode Statistics (HES), and the National Vascular Database as well as for longer term follow-up in the event the trial is extended.

Where can you find out more about how your information is used?

You can find out more about how we use your information

- at www.hra.nhs.uk/information-about-patients/
- by asking one of the research team
- by sending an email to DM-PAD@imperial.ac.uk, or
- by ringing us on 0203 311 5208.

Normahani P, *et al. BMJ Open* 2022; 12:e066950. doi: 10.1136/bmjopen-2022-066950

Complaint

If you wish to raise a complaint on how we have handled your personal data, please contact Imperial College London's Data Protection Officer via email at dpo@imperial.ac.uk, via telephone on 020 7594 3502 and/or via post at Imperial College London, Data Protection Officer, Faculty Building Level 4, London SW7 2AZ.

If you are not satisfied with our response or believe we are processing your personal data in a way that is not lawful you can complain to the Information Commissioner's Office (ICO). The ICO does recommend that you seek to resolve matters with the data controller (us) first before involving the regulator.

Involvement of the General Practitioner (GP) / Family Doctor:

With your permission, your GP and other doctors involved in your clinical care will be kept informed of your participation in the study, but otherwise all information about you and your treatment will remain confidential. We may contact your GP to obtain information about your health status if we cannot reach you.

What will happen if I lose mental capacity during the study period?

This is expected to be a very rare occurrence. If this did occur your doctor or carer will determine whether you should be withdrawn from the study. If you are withdrawn, any identifiable data already collected with consent will be retained and may be analysed, but no further data will be collected or any other research procedures carried out on or in relation to you.

What will happen to the results of the research study?

When the study is complete, we plan to inform patients of the results of the study by letter, email, newsletter, social media or publication on the trial website. We may ask patients if there are any other methods they would prefer. The results will be presented at conferences and published in a medical journal. No individual participants will be identified.

Who has organised, reviewed and funded the research and who will be supervising it?

This research has been supported by a National Institute for Health Research, Health Technology Assessment programme grant, which is funded by the National Institute for Health Research. The Sponsor of this study (Imperial College London) will pay your hospital to cover the costs of your participation in this study. You are able to claim the travel costs (e.g. bus / train / tube fare or parking costs and petrol) for your hospital visits. Please speak to the study nurse about how to make this claim.

The research is being co-ordinated by Imperial College London, who have overall responsibility for coordination of the study. The research has been reviewed by the National Institute for Health Research, representatives from all of the participating hospitals and organisations, and an independent National Research Ethics Committee, and the Health Research Authority (HRA).

Contact Details

If you have any further questions about your treatment, please discuss them with your doctor. You may also find it helpful to contact the research nurse on XXXXXX.

If you would like further information about clinical research, the UK Clinical Research Collaboration (a partnership of organisations working together on clinical research in the UK) has published a booklet entitled ‘Understanding Clinical Trials’. Contact UKCRC: website: http://www.ukcrc.org/wp-content/uploads/2014/03/iCT_leaflet.pdf

THANK YOU FOR READING THIS INFORMATION SHEET

DM PAD
A multicenter comparative diagnostic accuracy study

Delete this line, then print on Hospital/Trust headed paper

Site ID:	Initials:
Participant Trial ID:	Principal Investigator Name:

Diagnostic tools to establish the presence and severity of peripheral arterial disease in people with diabetes (DM PAD)

IRAS 301408

PATIENT CONSENT FORM Please initial box

1. I confirm that I have read and understand the information sheet dated 06/10/2021 (Version 2.0) for the above study and have had the opportunity to ask questions which have been answered fully. ☐

2. I understand that my participation is voluntary and that I am free to leave the study at any time without my medical care or legal rights being affected. ☐

3. I understand that relevant sections of my medical records may be looked at by authorised individuals from the research team, from regulatory bodies, from the study Sponsor, or from the NHS Trust in order to check that the study is being carried out correctly. I give permission, provided that strict confidentiality is maintained, for these bodies to have access to my medical records for the above study. ☐

4. I agree to my data being entered onto a secure database held at the University of Edinburgh, in accordance with the Data Protection Act 2018. ☐

5. I understand that my pseudonymised data will be transferred to the University of Granada for the analysis. ☐

6. I understand that my CTA or MRA scan images will be transferred to the University Hospitals of Leicester NHS Trust for reporting. ☐

7. I agree to my GP, or any other doctor treating me, being notified of my participation in this study. I agree to my GP being involved in the study, including any necessary exchange of information about me between my GP and the research team. ☐

Normahani P, *et al. BMJ Open* 2022; 12:e066950. doi: 10.1136/bmjopen-2022-066950

8. If during the study my clinical care team determine that I have lost capacity to provide informed consent, I will be withdrawn from the study and any identifiable data collected with consent would be retained and used in the study.

9. I agree to take part in the DM PAD study.

Optional consent section (please initial the appropriate box)

10. I give/do not give consent for information collected about me to be used to support other research in the future, including those outside of the EEA.

Give consent ☐ Do not give consent ☐

11. I give/do not give consent for my data may be linked with appropriate national databases, including Hospital Episode Statistics (HES), and the National Vascular Database as well as for longer term follow-up in the event the trial is extended

Give consent ☐ Do not give consent ☐

12. I give/do not give consent to be contacted in the future with regards to this study, should the study be extended.

Give consent ☐ Do not give consent ☐

Full Name of Participant	Date	Signature
Name of Person Taking Consent	Date	Signature

(1 copy for participant; 1 copy for the patient's medical notes, 1 copy for the site file)

Appendix 2- Details of index tests

Index tests

ABPI

ABPI measurements will be performed using a sphygmomanometer cuff placed at the ankle and a handheld audible CW Doppler device (Dopplex D900 Audio only Doppler, Huntleigh Healthcare Ltd., Cardiff) to measure dorsalis pedis and posterior tibial artery systolic pressure. Brachial artery pressures from both arms will be taken and the highest reading used to calculate the ABPI.

Exercise ABPI

Exercise ABPI traditionally requires a treadmill. This limits its use in primary care, where a treadmill is not available. Additionally, the results of the patient and public involvement (PPI) work suggest that 43% of patients will not be able to walk on a treadmill (due to disability, frailty or DFU) and that an additional visit to a vascular laboratory for this test would not be acceptable. To ensure patient acceptability, repetitive heel raising will be used. This can be performed in clinics, has excellent correlation with treadmill testing (26,27) and has been advocated by the American Heart Association (28). Our PPI focus group considered this test acceptable.

The exercise ABPI protocol, will consist of 50 consecutive repetitions of active dorsiflexion whilst standing (26). The knees should be kept fully extended. Participant will be allowed fingertip support against a wall to assist with balance. The protocol will be symptoms limited, so that premature termination of exercise will be permitted if the subject experiences lower limb discomfort, chest pain, shortness of breath or feels unwell for any other unspecified reason. Instances of premature termination, and accompanying reasons, will be recorded. ABPI will be measured using the same methodology as outlined above.

In some patients, exercise ABPI may not be possible due to deformities of the foot, e.g., those with forefoot amputation, Charcot foot syndrome and forefoot plantar ulceration. Foregoing exercise ABPI will be left to the clinical teams discretion. Reasons for foregoing exercise ABPI will be documented.

TBPI

Measurements will be made using the Huntleigh toe pressure kit (Huntleigh Healthcare Ltd., Cardiff) employing an infrared sensor placed on the hallux. The highest brachial upper limb reading will be used to calculate the TBPI.

Audible handheld Doppler

Audible CW Doppler interrogation of the dorsalis pedis and posterior tibial artery (Dopplex D900 Audio only Doppler, Huntleigh Healthcare Ltd., Cardiff).

Visual handheld Doppler

Visual CW interrogation of the dorsalis pedis and posterior tibial artery using the handheld Huntleigh Digital Dopplex device (Huntleigh Healthcare Ltd., Cardiff).

PAD-scan (in selected centres)

The PAD-scan will be performed using a portable ultrasound system (Mindray M7; Shenzhen, China) with a linear 6-14Hz transducer. The anterior tibial and posterior tibial artery will first be visualised at the ankle, using B-mode imaging and colour Doppler, in transverse and then longitudinal planes. Arterial spectral waveforms will then be sampled from the centre of each vessel using a Doppler angle of <60°. Waveforms will be optimised for interpretation by adjusting sample volume, sample size, Doppler scale, Doppler gain and wall thump filter settings.

Diagnostic thresholds

We will evaluate the performance of the index tests based on prespecified diagnostic thresholds for PAD. These thresholds have been selected as they demonstrated optimal diagnostic performance in the TrEAD study or are commonly used in clinical practice. However, other thresholds have been described in the literature and there is no consensus as to which are best. Therefore, we will evaluate different 'exploratory' thresholds as part of our secondary analyses. For tests generating continuous results (ABPI, TBPI and exercise ABPI) we will also evaluate performance based on optimised thresholds derived from Receiver Operating Characteristics (ROC) analysis. We will use a 'net benefit' approach (as a

sensitivity type analysis over a range of plausible thresholds) following ideas for assessing the clinical utility of prognostic models summarised in Riley R et al (*Prognostic Research in Health Care; 2018; Oxford; section 7.4.3 page 168-170*). From this, it should be possible to integrate cost-effectiveness parameters into assessing the best threshold.

Diagnostic thresholds:

- Visual waveform assessment- monophasic or biphasic waveforms with adverse features.
- Audible waveform assessment- monophasic waveform
- ABPI- ≤0.9 in either vessel
- TBPI- <0.75 in either vessel
- Exercise ABPI (31)- Post exercise ABPI ≤0.9 in either vessel.

Normahani P, *et al. BMJ Open* 2022; 12:e066950. doi: 10.1136/bmjopen-2022-066950

Appendix 3- Reference CTA and MRA protocol

CTA reference scan protocol

Peripheral CT angiograms can be obtained with all current multiple–detector row CT scanners (i.e., four or more channels). A standardised scanning protocol programmed into the scanner and the study can easily be performed in 10–15 minutes of room time. Breath holding is required only at the beginning of the CT acquisition through the abdomen and pelvis. A medium to small imaging field of view (with the greater trochanter used as a bony landmark) and a medium to soft reconstruction kernel are generally used for image reconstruction, imaging continues to the whole foot. Series 1 is imaged from Diaphragm to Ankles and then from the Knees to Ankles to achieve a delayed phase of imaging especially if there is a proximal stenosis/occlusion causing a delayed flow of contrast to the ankles. A region of interest is taken in the level of the descending thoracic aorta (DTA) or coeliac axis. A 10–15 mm2 circular region of interest is placed inside the middle of the aortic lumen and this will subsequently measure the Hounsfield units of the aortic lumen on subsequent scanning. At 10 seconds following IV contrast administration, serial low-dose monitoring CT scans are obtained at the same table position (DTA or coeliac axis level) at 2-second intervals. When the region of interest detects a pre-set contrast enhancement level (usually a 100– 150 HU value), there is automatic triggering of the scanner to acquire images in the desired scan range, usually from the level of the celiac axis to the feet. This time-efficient method ensures optimal arterial enhancement within the region of interest. In general, 75 mls contrast is used with a chasing bolus of 100 mls Saline to produce a compact volume load. This is injected at a rate of 4 ml/sec from a Venflon in the antecubital fossa. CT settings; kVp: 100-120, TI/pitch: 1.3/111 (fast) 0.8/27-65; FOV: 350-400(L), Rotation time: 0.35-0.5; SD: 12-12.5 (all standard sure exp), Detector configuration: 0.5x80 (depending on centres' CT scanner channel). This protocol allows reconstruction of the dataset on a 3D workstation, therefore all images will be able to be reconstructed at any plane by the reporting radiologist.

MRA reference scan protocol

MR angiography will be performed in the local hospitals scanner 1.5-3T MR. An AP phased array surface body coil will be used in conjunction with a standard receivers for signal transmission and reception. The coil is placed to cover the lower region of the abdominal portion of the aorta and included the iliac arteries to the level of the inguinal ligament. Coverage is from the diaphragm to the foot. Depending on the centre either a test bolus of Gadolinium based contrast agent is injected via the antecubital vein or to the infrarenal region of the abdominal aorta, which combines a test bolus with a multiphase, single section, gradient recalled echo sequence. A bolus tracking method will be obtained with a ROI in the aortic lumen at the level of the coeliac axis where repeat scanning is performed, the full bolus of contrast is injected at a rate of 2-3mls/sec from a Venflon in the antecubital fossa is administered, and when the bolus is detected within the vessel, the technologist can trigger scan acquisition. The coronal oblique plane is preferred for bolus-chase MRA because it covers the largest field of view in the shortest scanning time while maintaining high spatial resolution in the slice-select direction. Subtraction techniques will be employed to improve contrast resolution in CE-MRA.

Short TR and TE for fast acquisition are accomplished with 3D spoiled gradient-echo pulse sequences. Spoiling increases the contrast-to-noise ratio (CNR) by suppressing residual background signal. As in other MR applications, the acquisition time is determined by the TR, the number of phase-encoding steps, the number of slices, the fraction of k-space sampled, and the acceleration factor (when parallel imaging is used). The gradient strength governs the shortest possible TR (< 5 milliseconds) and TE (< 3 milliseconds), although parameters such as wider bandwidth, smaller flip angles, and fractional echo can shorten the TR and TE. A flip angle of 15-45° is typically used. MRA can be acquired with either a single phase or a time-resolved MRA, depending on the centres preference. Using these sequences, it provides anisotropic images, which allows reconstruction of the dataset on a 3D workstation, therefore all images will be able to be reconstructed at any plane by the reporting radiologist.

Normahani P, *et al. BMJ Open* 2022; 12:e066950. doi: 10.1136/bmjopen-2022-066950

Appendix 4- Angio score

(INSERT)

Appendix 5- Evaluation of patient acceptability Likert scale

**Overall, how satisfied were you with each of the tests you have had today?**					
	Very unsatisfied	Unsatisfied	Neutral	Satisfied	Very satisfied
Ankle-brachial pressure index (ABPI)					
Exercise ankle-brachial pressure index (Exercise ABPI)					
Toe-brachial pressure index					
Audible handheld Doppler					
Visual handheld Doppler					
PAD-scan (if applicable)					

Appendix 6- Study management structure

The study will be coordinated by a trial manager who will report to the Chief Investigator. The trial manager will liaise with local principal investigators to ensure that the trial is conducted locally according to protocol and in an expeditious manner. The organisational structure and responsibilities are outlined below.

Trial Management Group

A Trial Management Group (TMG) will be convened including the Chief Investigator, co-investigators and key collaborators, trial statistician and trial manager. The TMG will be responsible for day-to-day conduct of the trial and operational issues. Meetings will be held monthly throughout the set up and recruitment phase and alternate months subsequently until trial closure. Details of membership, responsibilities and frequency of meetings will be defined in separate terms of Reference.

Trial Steering Committee

A Trial Steering Committee (TSC) will be convened including as a minimum an independent Chair, independent clinician, the Chief Investigators and Trial Manager. A TSC meeting will be held at the start of the study prior to commencement of recruitment and at least annually as per NIHR guidelines. The role of the TSC is to provide overall supervision of trial conduct and progress. Details of membership, responsibilities and frequency of meetings will be defined in a separate Charter. A lay PPI representative will be included.

Data Monitoring Committee

A data monitoring committee meeting will be held prior to first patient first visit and will then be held prior to each TSC meeting. Further details will be defined in the separate DMC Charter. Statistical advice and analysis will be conducted by Professor John Norrie (ECTU), who has advised on this studies design and sample size. Professor Norrie will produce the Statistical Analysis Plan and subsequent reports for the Data Monitoring Committee.

Special advisory group

As successful primary (general practice & community) care recruitment is a priority, a Special Advisory Group (SAG) chaired by two leading experts in primary care diabetes and vascular medicine (Professor Kamlesh Khunti and Professor Azeem Majeed) will be formed. Other members of the SAG include, Ms Trusha Coward (community podiatrist), Ms Joanna Pitt (primary care nurse), Ms Caroline Durack (primary care manager), Dr Patrick Holmes (General Practitioner) and Professor Ahmet Fuat (General Practitioner).The SAG will advise on the recruitment and delivery of the study outside of secondary care to ensure that patients from these healthcare settings are adequately represented. Both chairs will sit on the TMG.

Patient Advisory Group

A Patient Advisory Group (PAG) will be convened. PAG will be meet annually prior to TSC meetings to ensure that a wide range of patient perspectives are considered during the study.

Early Discontinuation of the Study

There are no formal stopping rules but safety will be reviewed periodically by the DMC who could recommend early discontinuation of the study.

Risk Assessment

A study-specific risk assessment will be performed prior to the start of the study by the study sponsor. The risk assessment will consider all aspects of the study and will be updated as required during the course of the study. The risk assessment will consider all aspects of the study and will be updated as required during the course of the study.

Monitoring

The study will be monitored periodically by trial monitors to assess the progress of the study, verify adherence to the protocol, ICH GCP E6 guidelines and other national/international requirements and to review the completeness, accuracy and consistency of the data.

Monitoring procedures and requirements will be documented in a Monitoring Plan, in accordance with the risk assessment.

Normahani P, *et al. BMJ Open* 2022; 12:e066950. doi: 10.1136/bmjopen-2022-066950

Quality Control and Quality Assurance

Quality Control will be performed according to Imperial College internal procedures. The study may be audited by a Quality Assurance representative of the Sponsor. All necessary data and documents will be made available for inspection.

The study may be subject to inspection and audit by regulatory bodies to ensure adherence to GCP and the UK Policy Framework for Health and Social Care.

Peer review

This research has been reviewed by the Surgery Peer Review Board at Imperial College London, the DM PAD multicentre research group and the Collaborations Committee at Edinburgh Clinical Trials Unit. The scientific quality of the research was also reviewed and assessed by the NIHR HTA external reviewers as part of the grant application for funding, which was subsequently awarded.

Normahani P, *et al. BMJ Open* 2022; 12:e066950. doi: 10.1136/bmjopen-2022-066950
