# Supplementary material for: Study protocol for a multicentre comparative diagnostic accuracy study of tools to establish the presence and severity of peripheral arterial disease in people with diabetes mellitus: the DM PAD study
Source: BMJ Open. 2022 Nov 3;12(11):e066950. doi: 10.1136/bmjopen-2022-066950 (PMC9639108; doi:10.1136/bmjopen-2022-066950)
Supplement: Reviewer comments [file bmjopen-2022-066950.reviewer_comments.pdf]

## PEER REVIEW HISTORY

BMJ Open publishes all reviews undertaken for accepted manuscripts. Reviewers are asked to complete a checklist review form (**http://bmjopen.bmj.com/site/about/resources/checklist.pdf**) and are provided with free text boxes to elaborate on their assessment. These free text comments are reproduced below.

## ARTICLE DETAILS

| | |
|---|---|
| **TITLE (PROVISIONAL)** | Study protocol for a multicentre comparative diagnostic accuracy study of tools to establish the presence and severity of Peripheral Arterial Disease in people with Diabetes Mellitus: the DM PAD study |
| **AUTHORS** | Normahani, Pasha; Burgess, Laura; Norrie, John; EPSTEIN, DAVID; Kandiyil, Neghal; Saratzis, Athanasios; Smith, Sasha; Khunti, Kamlesh; Edmonds, M.; Ahluwalia, Raju; Coward, Trusha; Hartshorne, Tim; Ashwell, Simon; Shalhoub, Joseph; Pigott, Elizabeth; Davies, AH; Jaffer, Usman |

This article was not externally reviewed at BMJ Open. Protocols that have been independently assessed prior to submission to BMJ Open are usually fast-tracked to publication on the grounds that further substantial changes will not be possible. This independent assessment will usually be external, independent review for both a substantial grant award from a non-commercial or government funder and ethics approval.
